# The role of age and business coaching in the relationship of lean startup approach and innovative work behavior of women entrepreneurs during COVID-19

**DOI:** 10.3389/fpsyg.2022.946918

**Published:** 2022-08-30

**Authors:** Cui Na, Rimsha Khalid, Mohsin Raza, Edwin Ramirez-Asis, Rosario Huerta-Soto, Atif Jahanger

**Affiliations:** ^1^School of Business Administration, Ningbo Polytechnic, Ningbo, China; ^2^Faculty of Management Sciences, Phuket Rajabhat University, Phuket, Thailand; ^3^Faculty of Management Sciences, Phuket Rajabhat University, Phuket, Thailand; ^4^Academic Department of Administration, Universidad Señor de Sipán, Chiclayo, Peru; ^5^Academic Department of Economics, Universidad Cesar Vallejo, Lima, Peru; ^6^School of Economics, Hainan University, Haikou, China; ^7^Institute of Open Economy, Haikou, China

**Keywords:** women entrepreneurs, entrepreneurship, business coaching, lean start-up, COVID-19

## Abstract

The purpose of the startup approach is to find an appropriate course of action that adds value to the economy’s development. This study is aimed to determine the effect of the lean startup approach with mediating effect of business coaching to foster innovative work behavior in women entrepreneurs. Additionally, the study also examines the moderating effect of age on the lean startup approach and innovative work behavior. A quantitative approach was employed. The findings show that the relationship between the lean startup approach and innovative work behavior is significant. Moreover, the findings also revealed a significant mediating effect of business coaching and moderating effect of age. This research encourages practitioners and scholars to deal with women entrepreneurship incubation initiatives in the lean startup methodology. Furthermore, this study also leads to a deeper understanding of women’s ideas for business exploration, growth, and implementation. The study contends that guidelines and directives are critical for creative entrepreneurial behavior.

## Introduction

The role of women’s entrepreneurship is quite important in fostering the country’s economic development and social contribution. Likewise, to strengthen their financial position, many females are inclined to start their businesses to embrace innovative opportunities for growth (Khalid et al., 2020). This has attracted many scholars to investigate women’s entrepreneurship in literature (Cohen et al., 2019). The startup concept includes business creators presenting new-fangled products and creative ideas in the market (Paternoster et al., 2014). Therefore, Dorf and Blank (2012) recommend that startups should have a key objective of repeatable and expandable business patterns. Particularly, in the context of developing countries where economic conditions are unpredictable, so to compete with such unpredictable conditions business sectors are making ways toward improving and presenting new products and services.

For instance, women in Peru are more likely to start a business. Peruvian women own and run a higher percentage of companies than in other Latin American countries. In Peru, Small and medium enterprises are about 98% of total business establishments, and 40% are women-owned enterprises (Nestsiarchyk, 2016). Women entrepreneurs contribute to Peru’s economy, providing millions of jobs in impoverished communities and sustaining domestic social growth. Concerning this, the role of women’s entrepreneurship in developing the country’s economy is equally important as men (Olah et al., 2021). Peruvian women are still restricted from starting their businesses and they need appropriate business training, according to a growing body of study, particularly in the solar sector (Glemarec et al., 2016). However, despite the significance of women entrepreneurs, they are still not involved in entrepreneurial activities and innovative behaviors (Giones et al., 2020; Vaka et al., 2020; Lambovska et al., 2021).

Following the arguments mentioned above, there is a need to implement some novel approaches as innovative work behavior boosts the confidence and motivation of women entrepreneurs to develop their startups/businesses. Until now, female entrepreneurs are also limited to business programs that assist them in characterizing their concepts and ideas.

Typically, entrepreneurial fermentation initiatives have focused on market development strategies and organizational framework enhancement. This framework is useful for developing education and various service offerings. Moreover, as much as these market methods are concerned, entrepreneurs must take creative ways of reaching out to prospective buyers to ensure innovative work behaviors in business (Ben Salem and Lakhal, 2018).

The strategy of a lean startup is critically important. Furthermore, there is research on entrepreneurs; therefore, to fill the gap, this research explores the role of female entrepreneurs in the context of lean startups. However, coaching/training is not included in the strategy of lean startup; it also represents the effect when the knowledge is transmitted to female entrepreneurs *via* various methods of business coaching. Thus, as a result of entrepreneurial coaching, the position of innovative, development-promoting, and helpful coaches will change from trainers to knowledge providers. In addition, coaching in business plays an important role in implementing the business plans from their ideas and then evaluating them with a lean startup approach (Paina et al., 2017).

The strategy of the lean startup is demonstrated by new ways to ensure useful procedures and policies for entrepreneurs. It offers a wider way of thinking and initiating business. In addition, a unique setting in which coaches build creative entrepreneurs is provided by a lean startup approach (Dutt et al., 2016). On a similar note, the lean startup approach is critical in establishing the course of action or guideline procedures for the workshops, mentors, and lecturers offering services to startup entrepreneurs. These guiding procedures assist female entrepreneurs in improving their organizational structure and methodologies for conducting business sessions (Mansoori et al., 2019). Moreover, such coaching and training sessions usually line up with certain aspects, such as assumption development and validity, collaboration with customers, and data analysis.

In addition, methodology procedures are still part of the teaching/coaching, leading to regularity and anomalies in the coaching process (Pauwels et al., 2016). On the same note, the literature review emphasizes the significance of innovative work behavior in the context of the lean startup approach which is linked with business training for female entrepreneurial behavior, even though in the review of related studies, the significance of entrepreneurs coaching has already been highlighted. Furthermore, the previous studies established the presence of coaching, while excluding the introduction of a systematic review for coaching activities with entrepreneurs’ relationships (Adomdza, 2016).

Likewise, when it comes to adopting innovative behavior, the age factor cannot be ignored as it has an important association with a direct or moderating role (Wang et al., 2009; Chung et al., 2010). Age gaps influence the difficulty of learning new methods (Morales and Marquina, 2009). Furthermore, the younger generation is less anxious concerning new learning skills than older generation people, and low learning anxiety levels are associated with a greater willingness to try new things. To accomplish the objective, the study suggested the following purposes. First, this study examines the impact of lean startup approaches on creative work behavior. The second objective then examines how the lean startup approach influences business coaching. The third objective is to investigate the impact of business coaching on innovative work behaviors. Fourth, this study is also intended to measure the mediating effect of a business coach on the lean startup approach and innovative work behavior. Besides, this research is also inclined to determine the moderating role of age between the lean startup approach and innovative work behavior.

The significance of female entrepreneurs has grown as social enterprise activities begin to focus on females in the energy services sector. Women assume that they will have fewer opportunities and a difficult time finding buyers. Entrepreneurial initiatives encourage females to pursue careers in the energy business due to the mutually beneficial capability of various combined goals for instance providing new opportunities in education and health for family interests and extension of female monetary development (Tirumalsety and Gurtoo, 2021). Derived from the objectives, four research questions were formulated as indicated below:

The research questions of this study represent how the practice of lean startup influence women’s innovative work behavior in business. Second, how does lean startup affect business coaching? Third, does business coaching affect innovative work behavior? What impact does a lean startup have on the innovative work behaviors of female entrepreneurs as a result of business coaching? In addition, how does age influence the lean startup strategy and innovative work behavior?

This study has significance by executing the lean startup methodology to provide business coaching to women entrepreneurs in the solar energy sector of Peru, intending to develop innovative work behavior. It improves understanding of the coach’s responsibilities and training sessions regarding development and content.

## Literature review

### Innovative work behavior in women entrepreneurs

Innovative work behavior is defined as the purposeful creation, introduction, and implementation of new ideas within the organizational objective that benefits the business’s performance (El-Kassar et al., 2022). A business enterprise means the ability to make ideas, turn them into products and services, and then sell them (Johnson and Sohi, 2001). Business operations play a crucial role in boosting the economies of developed countries (Vasudevan and Paralkar, 2016). Bruni et al. (2004) show the perspectives they provided to create a woman business model. Their concept included a variety of situations, such as a young woman who starts her own company to avoid uncertainty, a female whose career is associated with a business due to a family tradition, and a young woman who has a clear idea of her job or business to run. Furthermore, the model included a woman starting a company in the world that does not give enough reorganization while running a small business, further a woman who may have quit her job or profession to focus on family responsibilities and some women who return to a company for their identity or financial purposes (Khalid et al., 2021; Valaskova et al., 2021b).

Furthermore, products have a shorter life span in rapidly changing environments, affecting market growth. As a result, organizations must implement innovative strategies by introducing novel products and services in a limited time frame. For businesses, creative thinking and transmitting those innovations’ ideas into beneficial goods and services are critical (Huamán et al., 2022). In fulfilling the need for growth and development, the shortage of solar energy is a major problem in developed countries (Gray et al., 2019). Energy shortage is described by Bouzarovski (2014) as a family’s way of managing, protecting, or connecting sufficient energy resources. Without the contribution of women, a country’s economic advancement is impossible. Therefore, supporting and strengthening women’s contributions has become important to a country’s growth (Ismail, 2018).

Researchers, such as Clancy et al. (2007), argue that the gender-based energy scarcity concept has been excessively overlooked. Although energy shortage affects women and men, the impact of home energy production is unequally felt by young females especially those who face low-income and developing countries owing to conventional socio-cultural factors. It affects females’ wellbeing, as females are more likely to be exposed to smoke in poorly ventilated households because women are typically responsible for home activities (Valdivia, 2015).

Innovative work behavior, on the other hand, would promote creativity in the workplace by leveraging collaboration to formulate and implement creativity interventions (Pratoom and Savatsomboon, 2012). Considering innovative job activity, typically starts from solutions that stem from seeing possibilities and challenges that need to be addressed (de Waal and Maritz, 2022). Exploring solutions entails searching for innovative approaches or devising new methods for producing similar goods and services more efficiently (Durana et al., 2021b). Researchers, such as Dorenbosch et al. (2005), argued that generating ideas is usually about the precision of novel solutions and innovative thoughts for risky problems. Preparing support, convincing, and supporting substantial hierarchical individuals with new concepts is referred to as champing of the concept (Madrid-Guijarro et al., 2016). Finally, the idea’s execution is defined as transforming creative concepts into tangible results. Caniëls and Rietzschel (2015) defined the concept of implementation as “the creation of innovative goods, facilities, and the implementation of ideas within the market.” Implementing new technology, computer use, and e-practices have provided the development in business innovation in developed countries (Khan et al., 2012). In developed countries, female entrepreneurs in the solar energy field strongly need innovative job behavior to alleviate poverty.

### The lean startup approaches

The lean startup model requires experimentation when exploring opportunities. Slavik et al. (2020) support strategic decision-making, and argue that efficient searching and iterative planning of events are unstructured. This study includes the lean startup strategy, entrepreneurship consulting, and women’s innovative job behavior startups.

Lean production promoted the lean startup approach. Lean production reduces waste and increases resource efficiency (Ries, 2011). “Disciplined Entrepreneurship,” “discovery-driven strategy,” and “probe and learn” are some of the concepts in this methodology (Sull, 2004; Williams et al., 2021). This technique assists in integrating various approaches and philosophies, such as model design to think, customer framework creation, and concepts for software development (Dorf and Blank, 2012). The most critical aspect of this approach is that it supports learning through a series of well-designed trials (Chengbin et al., 2022). In terms of functional implications, these tests involved acceptable failure and elasticity with practical implications (Frederiksen and Brem, 2017; Matkevičienė and Jakučionienė, 2021). The lean startup methodology is based on a formal, strict information system and a distinct vocabulary (Krulicky and Horak, 2021; Zhang et al., 2021). This technique, on the other hand, can be viewed as a symbolic boundary that encourages people to use the approach to show their competencies (Koskinen, 2005). The testing of framed hypotheses will help to improve accuracy in learning. As a result, individual consumers can do an analysis of reliable scientific statistics (Lǎzǎroiu et al., 2021; Valaskova et al., 2021c).

The lean startup approach is a returning process that includes three operations forms. Entrepreneurs break their concepts into definitive hypotheses that steer into a functioning business plan as testing assumptions (García-González and Ramírez-Montoya, 2021). This pictorial representation encompasses all facets that can be positively verified about that particular entrepreneur. The entrepreneurs lead the second process, which consists of testable hypotheses that are related to their business concepts (Ries, 2011). As a result, the criticality of the results can be shown for the running phase, which includes a series of evaluations.

In addition, entrepreneurs make long-term decisions on what would happen as they engage with specific consumers. The core principle of the lean startup strategy is to determine which product is best suited for the business. This data relates to a product’s concept in the industry and how the buyer receives a lot of money in exchange for it (Dorf and Blank, 2012).

Entrepreneurs assess the idea as part of their method. The research illustrates that entrepreneurs are designers who incorporate imaginary opportunities into equations and predictions based on the firm’s analytical viewpoint.

The iterative aspect includes theorizing, evaluating, using input from cognitive change assessments, and rehashing the method before the businessperson arrives at an adaptable course of action. However, as a result, two concerns arise when thinking about the lean startup as an iterative method (Crane, 2021). The main issue is about the elements of this method used to put the lean startup principle into practice. As a result, what are the actions that projects take if they use the lean startup to explore startup opportunities? Second, if we understand these gestures better, we will see how they are linked to venture execution. As a set of operations, the lean startup might be considered a skill (Harms and Schwery, 2020). As a result, this skill will assist entrepreneurs in developing more accurate predictions regarding a promising business venture (Felin and Zenger, 2017). Furthermore, it enables market visionaries to generate these hypotheses faster by assisting them in fabricating knowledge, developing traditional operating plans, and increasing self-confidence (Bakker and Shepherd, 2017; Thomas et al., 2012). The actual goal of this process is to provide women entrepreneurs with feedback on the actual findings of the business model. This research begins by illustrating woman entrepreneurs, who are then used for investigation. Furthermore, this research develops and explores the operationalization of their groundbreaking ideas and a consideration of their functional consequences. The following hypothesis was tested in this study:

***H1:** The relationship between a lean startup approach and innovative work behavior is positive.*

### Business coaching

Coaching is mostly concerned with specialized training for entrepreneurs. Coaches also have answers to particular challenges and help people gain the ability to solve the issues that arise (Van Weele et al., 2017). Entrepreneurial coaching is a helpful platform for entrepreneurs who want to advance their entrepreneurial skills. Furthermore, coaching is a collection of techniques that help entrepreneurs who lack the necessary expertise, experience, or awareness to launch their businesses. Furthermore, it would assist entrepreneurs with entrepreneurial solutions. The relationship between an entrepreneur and a coach is determined by the entrepreneur and the coach’s realistic life experiences. The influence of equity, relationship with coaching, social learning, productive collaboration, and techniques employed in a progressive program are some of the variables that influence the success of the relationships between the businessmen and the coaches (Cox et al., 2014; Durana et al., 2021a). To promote confidence and transparency with entrepreneurial coaching, it is essential to defy hierarchy (Hampel et al., 2020). Furthermore, conversations may be facilitated by activities, such as listening, explaining, encouraging contemplation, and asking. Additionally, company coaching aids the development and learning of ownership (Kliestik et al., 2020; Valaskova et al., 2021a). The stage that offers the informative dialog with entrepreneurs is a central defining characteristic of the evolutionary coaching aspect (Brockbank and McGill, 2012). Define coaching is one-on-one assistance provided to a company visionary or a growth agency to meet a particular requirement to acquire, progress, or enhance skills (Khalid et al., 2021).

It is confirmed that coaching is a multifaceted concept that includes various types of instructing, such as mental, skilled, and enthusiastic mentorship, as well as the participation of a variety of entertainers, such as coaches, staff, and supervisors (Bayar et al., 2020; Karlsven, 2021). Furthermore, some researches focus on the teaching system, contending the concept of the association between performers and the styles of coaching that distinguishes informal from formal coaching (Rocchi et al., 2013). Formal coaching, for example, involves conventions, order, and planning, while informal coaching represents the acknowledgment of performers (Rajasinghe et al., 2021). In an informal organization, passion, and mental preparation are required as much as technical training in a structured association (Raza et al., 2020).

The procedures support the concept that differentiation, as well as performers, are important. Furthermore, combined training is formalizing and attempting to achieve mutual goals through informal programming that involves individuals identifying particular problems or meeting specific needs (John and West-Leuer, 2013). A few researchers focus on instructive measurements and entertainers, so advisors, successors, and material are essential (Devine et al., 2013). In the literature, there is no mention of a successful mentor’s judgment. As a result, according to a few researchers, decision-making is based on a set of specific parameters, including mentors’ skills and knowledge, learning a skill, and collaborative capability (Devine et al., 2013). In the advancement act, for example, the coach is an expert, or sociologist who can address societal and personal problems. Coaches are often industry visionaries, leaders, or individuals who indicate a temporary or lasting need (Myers et al., 2005).

Despite various unavoidable problems throughout the transmission period, the coaches mentioned above are the successors (Botha and Taljaard, 2021). While the research does not list uniform characteristics of coaches, it describes them as attentive, looking to use plans and tactics, and disbelieving to indicate that lack of trust, education, and training are elements of coaching requirements (Laschober et al., 2013). While the coach and successor selection is based on such criteria and conditions, the coach’s sympathy often determines the consistency of the step, even though the instructing norms may be established by both the relevant performer to the organization’s and performers’ needs and motivation (Shankar et al., 2020).

Coaches believe in an evolutionary strategy that engages resources in encouraging thought about women’s innovative job habits. In coaching, the lean startup approach is regarded as beneficial. Except for women entrepreneurs, reflecting on these ideas assists in understanding and improving the market practices. The following hypotheses were tested in this study:

***H2:** The relationship between lean startup approach and business coaching is positive.*

***H3:** Business coaching positively influenced innovative work behavior.*

***H4:** The relationship between a lean startup approach and innovative work behavior has been mediated by business coaching.*

### Age

A previous study recommended that age is a significant factor that has a significant direct and moderating relationship with behavior (Chung et al., 2010). The study by Chung et al. (2010) found that age as a mediator is a more powerful variable in explaining behaviors. Moreover, Morris et al. (2005) revealed that age is an important variable as a moderator when it comes to the adoption of behavior. In this study, age has a strong moderating relationship toward behavior with younger employees.

Likewise, age significantly affects learning acceptance (Chafloque-Cespedes et al., 2021). In another study, Czaja and Schulz (2006) suggested that old people possess low self-efficacy for learning. The study also found that old people think that it is not easy to learn new technology (Boulton and Turner, 2006). Likewise, some earlier studies mentioned that age differences could influence the perceived difficulty of learning new knowledge (Morris et al., 2005). Younger people have low anxiety about learning new skills as compared to older ones and low learning anxiety is linked with less hesitation to engage in new learning opportunities (Saunders and Levine, 2004; Chaffin and Harlow, 2005). Therefore, this relationship is not investigated in lean startup and innovative work behavior context. Regarding this study, it is assumed that the effect of age between lean startup and innovative work behavior will be stronger for young women entrepreneurs as compared to the older ones. Therefore, we propose the following hypothesis:

***H5**: The relationship between lean startup and innovative work behavior has been moderated by age.*

### Organizational creativity theory

Collaboration of people in an uncertain system may shape important programs, strategies, or procedures, which can be considered organizational creativity (Woodman et al., 1993). In the context of this study, innovation is described as the workplace at least one individual comes up with fresh and useful ideas.

A lean startup is a technique and approach that entrepreneurs may use to produce their products faster at a lower cost. It is predicated on the possibility that company visionaries would formulate such ideas regarding their venture and test them in a particular sector. On the other hand, these definitive conclusions may be placed to the test in the modern world. These goals are to disprove or confirm these hypotheses, as well as for a better understanding of how another activity works. Lean startup is a philosophy for developing learning defined as an ongoing shift in skills and knowledge (Tegano, 1990). It is most likely a case of active learning, in which industry visionaries improve by putting their theories to the test in real-world scenarios. Lean startup becomes a case of gathering-based active learning in modern pursuit classes.

**FIGURE 1:**
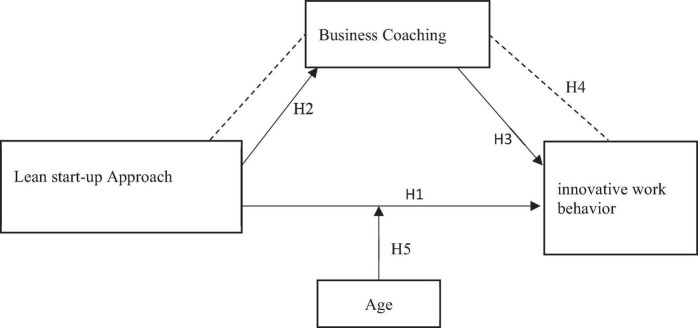
Theoretical framework.

Due to the technological and creativity advancements executives’ researchers can understand that lean startup includes disciplined entrepreneurship, lean startup, hypothesis-driven business, test, and learn (Blank, 2013). These methodologies emphasize on the importance of the early client communication, reflecting creativity and learning pace in a technical transition. It extends the applicability of lean startup to modern dares to grow companies and reduce fuzziness in front of technology (Stevens, 2014). Lean startup is now a system of enterprise education, as well as a practice used by an ever-increasing number of industry visionaries around the globe (Blank, 2013). The previous studies have shown that organizational creativity theory is a gathering built on active learning which represents an environment where marketers develop experience and expertise in the industry to demonstrate what business innovators have to know and what they have to do (Ladd et al., 2015). In this study, organizational creativity theory is applied in the sense of a lean startup approach. The learning procedures may be linked to a person and clear assessment of market knowledge, as well as a group-based developmental measurement of the authority of skills. Figure 1 shows the theoretical representation of study.

## Methodology

The study looks at woman entrepreneurs’ creative job behaviors and lean startup approaches. Furthermore, to look at the role of business coaching in mediating between the learning startup strategy and IWB, as well as moderating the role of age between variables. A formal questionnaire was used to obtain data in this analysis, which was done using a quantitative approach. The population of the study is Peru and the sample for data collection in this analysis is women entrepreneurs in the solar energy field in Lima, Peru. The convenient sampling technique was used in this study.

The structure of the questionnaire was based on a previous research. In specific, the lean startup method was modified using a 5-point Likert scale. Items were adapted from business coaching (Matlay et al., 2012). The IWB items were adapted from Kleysen and Street (2001). The data was collected using convenience sampling. Furthermore, 250 questionnaires were sent to women entrepreneurs by email. However, only 200 people responded. Therefore, the return rate of questionnaire distribution was 80%.

## Data analysis and discussion

**FIGURE 2:**
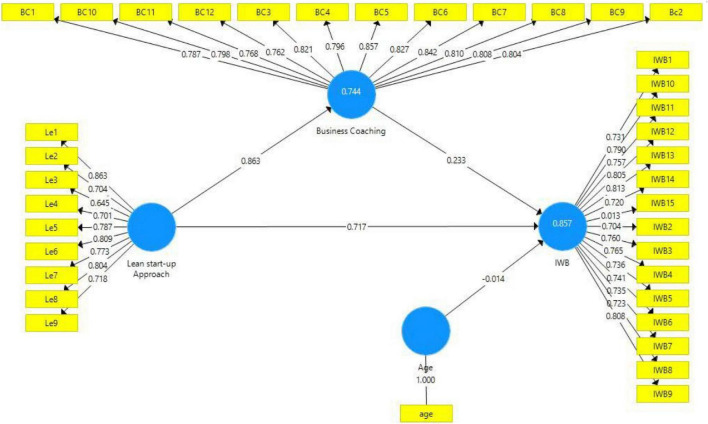
Measurement model.

Smart PLS, version 3.3.2, was used in this study. Smart PLS helps the researcher interpret the research participants’ responses to get a reliable and cohesive conclusion. The partial least square is used in this study by applying structural equation modeling (Hair et al., 2016). Figure 2 shows the measurement of model.

Demographic data shows that 52-woman entrepreneurs are single, 70 are engaged, 47 are divorced, and 31 are widows. Furthermore, 28 women are between the ages of 20 and 25. However, 36 females are between the ages of 26 and 35, 64 women are between the ages of 36 and 45, and 73 women are between the ages of 46 and 55. Moreover, 81 have received an elementary education, 52 have received a certificate or diploma, 36 had bachelor’s degree qualifications, and 31 have received higher education.

This result demonstrated the four variables in the study. The independent variable is the lean startup approach; the dependent variable includes IWB, moderating role of age, and mediating role of business coaching.

In Table 1, the outer loadings should be above 0.70 so that it is reliable and valid. Relatively, below 0.40 will be removed (Tabassum et al., 2020). All values are above 0.70, so, no reliability and validity issues were detected in this study. The Cronbach’s α, CR should be above 0.70 (Sarstedt et al., 2019). All values are greater than 0.70 in this study. Furthermore, AVE should be above 0.50. All values are greater than 0.50.

**TABLE 1:** Internal consistency.

	Loadings	Cronbach’s α	CR	AVE
Le1	0.863	0.906	0.924	0.576
Le2	0.704			
Le3	0.645			
Le4	0.701			
Le5	0.787			
Le6	0.809			
Le7	0.773			
Le8	0.804			
Le9	0.718			
BC1	0.787	0.951	0.957	0.651
Bc2	0.804			
BC3	0.821			
BC4	0.796			
BC5	0.857			
BC6	0.827			
BC7	0.842			
BC8	0.810			
BC9	0.808			
BC10	0.798			
BC11	0.768			
BC12	0.762			
IWB1	0.731	0.930	0.942	0.535
IWB2	0.704			
IWB3	0.760			
IWB4	0.765			
IWB5	0.736			
IWB6	0.741			
IWB7	0.735			
IWB8	0.723			
IWB9	0.808			
IWB10	0.790			
IWB11	0.757			
IWB12	0.805			
IWB13	0.813			
IWB14	0.720			
IWB15	0.013			
Age	1.000	1.000	1.000	1.000

### Discriminant validity

The discriminant validity is shown in Table 2. The HTMT method is more appropriate to calculate the discriminant validity. The HTMT values should be <0.85 (Hair et al., 2019). In this study, the HTMT values are below 0.85.

**TABLE 2:** The HTMT discriminant validity.

S. No.		1	2	3	4
1	Business coaching				
2	IWB	0.026	0.044		
3	Lean startup approach	0.306	0.490	0.033	

The regression analysis is seen in Table 3. The variance proportion of variables is measured by *R*^2^. To demonstrate a close association between variables, the value is higher than 0.70. The *R*^2^ value of business coaching is 0.499, indicating a positive mediating partnership between lean startup and IWB. Furthermore, the association between the lean startup strategy and IWB can be explained by the moderation impact, which has a value of *R*^2^ of IWB factor (0.421).

**TABLE 3:** Regression analysis.

	*R* ^2^	*R*^2^-Adjusted
Business coaching	0.499	0.497
IWB	0.421	0.417

Furthermore, the *R*^2^ adjusted meaning demonstrates the model’s comparison with various numbers of predictors. For a strong predictive model with the inclusion of a new component, it should be 0.70. By mediating impact, business coaching with an *R*^2^ adjusted (0.497) predicts a change in the paradigm in the partnership between the lean startup approach and IWB. Furthermore, the IWB of *R*^2^ adjusted (0.417) predicts a change in the model in the association between lean startup and IWB.

**FIGURE 3:**
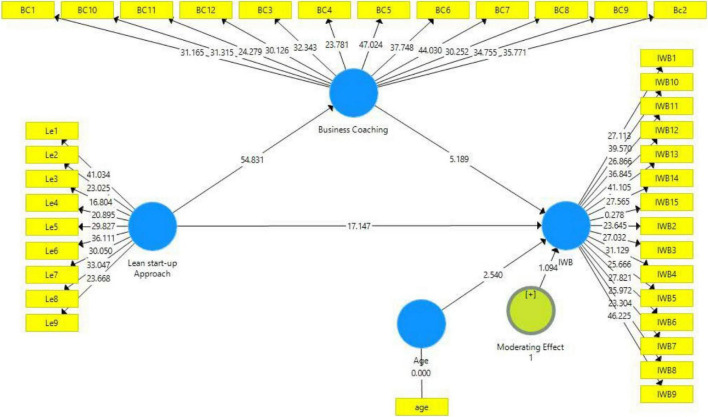
Structural equation model.

Table 4 shows that the *T*-value is above 1.96 and the *p*-value is below 0.05 that demonstrating the significance of the hypotheses. The results indicated that all *t*-values are above 1.96 and *p*-values are less than 0.05. There is a significant relationship between all variables and also a significant moderating effect of business coaching and the moderating effect of age between lean startup approach and innovative work behavior. Figure 3 displays the structural equation model.

**TABLE 4:** Direct and indirect effects.

	Coefficient	SD	*T*	*p*
Age - > IWB	0.053	0.021	2.540	0.011
Business coaching → IWB	0.234	0.045	5.189	0.000
Lean startup approach → Business coaching	0.863	0.016	54.831	0.000
Lean startup approach → IWB	0.714	0.042	17.147	0.000
AGE*Lean startup approach → IWB	−0.023	0.021	1.094	0.000
Lean startup approach → Business coaching → IWB	0.202	0.039	5.170	0.000

### Discussion

The study found the significant relationship between age and innovative work behavior (β = 0.053, *t* = 2.540, *p* = 0.011). The study is matched with the relationship between age and IWB. The previous study shows a significant effect on IWB (Wang et al., 2009).

This study found a positive and significant relationship between the lean startup and IWB [β = 0.714, *t* = 17.147 (>1.96), *p* = 0.000 (<0.05)]. The study matched the lean startup effects on coaching and how the development of these associations supports learning among business visionaries. The business coaching techniques also give power to the assumption evolving and transformative (Mansoori et al., 2019).

Furthermore, the lean startup has a positive and significant association with coaching [β = 0.863, *t* = 54.831 (>1.96), *p* = 0.000 (<0.05)] which supports the concept that the lean startup is a significant technique by entrepreneurs.

However, the coaching found a positive and significant association with IWB [β = 0.234, *t* = 5.189 (>1.96), *p* = 0.000 (<0.05)]. The association demonstrated higher values in the development of change and learning. The important attribute of innovative coaching is providing a deep discussion with entrepreneurs (Brockbank and McGill, 2012). The results indicate positive relationships among the variables which indicate that all hypotheses are accepted.

#### Moderating effect of age

This study examined the moderating role of age between lean startup and innovative work behavior. This study shows that there is a negative moderating role of age between the lean startup approach and innovative behavior (β = –0.023, *t* = 1.094, *p* = 0.000). The studies demonstrated that age has an important role in adopting behaviors. Researchers found a strong moderating effect and this suggests the lack of knowledge and lack of confidence in the workplace may show more barriers to use the older as compared to younger ones. The relationship was stronger.

#### Mediating effect of business coaching

This study examined the mediating role of business coaching between lean startup and innovative work behavior. The results (β = 0.202, *t* = 5.170, *p* = 0.000) show that there is a stronger mediating relationship between lean startup and innovative work behavior. However, the business coaching method also empowers the coaching to be transformative and assumption evolving (Brockbank and McGill, 2012; Mansoori et al., 2019).

### Theoretical and practical implication

This study focuses on the significant role of business coaching with a lean startup approach to designing coaching programs. It helps to increase the innovative behavior of women entrepreneurs. When planning activities, the accelerators need to combine different entrepreneurial coaching and experience theories for the business plan. This coaching and women entrepreneurs’ relationship is important for entrepreneurial development because they persuade the women entrepreneurs to take action in business to introduce entrepreneurial innovation especially among the older ones as compared to the younger ones. This study found that age has an important factor in developing innovative work behavior. Online coaches’ experiences are full of expert guidelines and their opinions on further achievement. Therefore, the lean startup effect business coaching for women entrepreneurs. This methodology helps experts understand how they can direct women entrepreneurs to explore innovative ideas and get useful ideas from exploration. Further, this methodology helps innovative idea building and implementation in actual as an entrepreneurial activity. However, the lean startup methodology leads the challenges in planning the entrepreneurial incubation programs through business coaches.

In this regard, the coaches are considered the knowledge and skills development facilitators rather than commanding broad experience elements. Coaches should restrict their authoritative suggestions and advice and permit women entrepreneurs to initiate business innovation. Therefore, they must provide the formulating opinions in subject to valid assumptions except for the past experiences. Further, it may be beneficial in distributing the functions of organizers and coaches. In the accelerator programs, coaches are the organizers in which some entrepreneurs share valuable coaching experiences more than the collection of data from the customers.

The study also provides theoretical implications for researchers with the value-added concept in literature. The study provides the enriching literature related to lean startup, business coaching, innovative work behavior, and age factor. Therefore, this research also offers new ways to explore the concept of women’s entrepreneurship globally.

## Conclusion

This study mainly focused on the significant role of entrepreneurial coaching between lean startup methodology and innovative work behavior of women entrepreneurs in Peru.

This study also examined the moderating effect of age on innovative work behavior, like how elderly and young generations adopt different behaviors.

The results of this study indicate that the lean startup approach/methodology substantially affects innovative behavior and business coaching. This approach also controls the organization and the quality of the content for coaching sessions and serving as a starting point for shared discussion. The lean startup method often instills confidence, motivation, and valuable collaboration with mentors, assisting female entrepreneurs in changing their thought patterns. Additionally, this strategy facilitates the progressive coaching process that aims at transforming older women entrepreneurs who cannot embrace modern technologies and information. This result contrasts with the direction of entrepreneurial coaching, which leans more toward a functional style. Coaches, on the other hand, do not seem to be impartial facilitators to guide women entrepreneurs following any technique, which contradicts the ideas of the lean startup strategy. Comparatively, authoritative recommendations have been considered to be more valuable than data. Lastly, coaches must be organized in a vicarious manner that can rapidly enhance female entrepreneurs’ knowledge and influence their interpersonal relations.

Because of its importance, the study recommends lean startup technique directions in coaching sessions and investigates the impact of this strategy on the organizational structure. The study will also help female entrepreneurs to know the business skills required to begin their companies. They should think of a creative way to increase business and understand measurement and performance assessment to achieve the desired results. Additionally, institutions should follow the lean startup teaching approach from an educational point of view. It assists policymakers in both developed and developing countries in making policies that facilitate the growth of entrepreneurial skills.

### Limitation and future research

This study has several limitations. The study is focused on female entrepreneurs. The future studies need to focus on different coaching styles concerning entrepreneurial incubation programs and the future research could also be done on how innovative work behavior influences male entrepreneurs.

Moreover, between lean startup and innovative work behavior, age as a moderator has been used in the study. However, other important factors such as marital status and experience need to be studied as a moderator in future studies. In addition, in the solar energy industry, the quality of the procedures should be assessed in conjunction with other business characteristics of entrepreneurial growth.

Moreover, this research observes women entrepreneurs’ creative/innovative behavior, but more research should be conducted on male entrepreneurs in solar energy companies. Additionally, future studies should explore the impact of lean startup and other entrepreneurial techniques on vicarious approaches and experimental learning. Likewise, studies are needed on the most effective and detailed online coaching approaches and techniques for fostering women’s entrepreneurship in the context of the COVID-19 disease outbreak. This study has adopted a quantitative approach, while additional research on women and men entrepreneurs using advanced mixed methodologies should be performed in the future. This research focuses on the development of economic activities across national borders; so, it should be investigated to see if the enterprise could be expanded globally.

## Data availability statement

The original contributions presented in the study are included in the article/supplementary material, further inquiries can be directed to the corresponding authors.

## Author contributions

All authors participated in the analysis and developed the manuscript.
